# The Mortality-to-Incidence Ratio Is Not a Valid Proxy for Cancer Survival

**DOI:** 10.1200/JGO.19.00038

**Published:** 2019-05-09

**Authors:** Libby Ellis, Aurélien Belot, Bernard Rachet, Michel P. Coleman

**Affiliations:** ^1^ London School of Hygiene and Tropical Medicine, London, United Kingdom

## Abstract

**PURPOSE:**

The ratio of cancer mortality and cancer incidence rates in a population has conventionally been used as an indicator of the completeness of cancer registration. More recently, the complement of the mortality-to-incidence ratio (1-M/I) has increasingly been presented as a surrogate for cancer survival. We discuss why this is mistaken in principle and misleading in practice.

**METHODS:**

We provide an empirical assessment of the extent to which trends in the 1-M/I ratio reflect trends in cancer survival. We used national cancer incidence, mortality and survival data in England to compare trends in both the 1-M/I ratio and net survival at 1, 5, and 10 years for 19 cancers in men and 20 cancers in women over the 29-year period from 1981 to 2009.

**RESULTS:**

The absolute difference between the 1-M/I ratio and 5-year net survival for 2009 was less than 5% for only 12 of the 39 cancer/sex combinations examined. For an additional 12, the 1-M/I ratio differed from 5-year net survival by at least 15%. The comparison is also unstable over time; thus, even when differences were small for 2009, the difference between 5-year net survival and the 1-M/I ratio had changed dramatically for most cancers between 1981 and 2009.

**CONCLUSION:**

The 1-M/I ratio lacks any theoretical basis as a proxy for cancer survival. It is not a valid proxy for cancer survival in practice, either, whether at 5 years or at any other time interval since diagnosis. It has none of the useful properties of a population-based survival estimate. It should not be used as a surrogate for cancer survival.

## INTRODUCTION

Population-based cancer survival is an essential public health indicator. Estimates of cancer survival are derived from population-based cancer registries that record a basic data set about each person resident in the territory covered by the registry who is diagnosed with cancer, in due course including details of the patient’s death. Together with cancer incidence rates, also derived from cancer registries, and cancer mortality rates derived from death registrations, these three measures comprise an invaluable tool kit for cancer control.^1^

In 1976, the ratio of the number of deaths attributed to a given cancer in a given population and the number of patients registered with the same cancer in the same year in the same population was proposed as a measure of the completeness of population-based cancer registration, in *Cancer Incidence in Five Continents, Volume III*, published by the International Agency for Research on Cancer.^2^ It was originally described as “deaths in period”, but later became known as the mortality-to-incidence ratio (M/I). The number of deaths had to be obtained from a source independent of the cancer registry. Where the number of cancer deaths exceeded the number of registered patients, cancer registration was considered likely to be incomplete. The M/I ratio has been widely used as a data quality indicator in cancer registries for more than 40 years.

In 1993, in *Cancer Incidence in Five Continents, Volume VI*, the M/I ratio was noted as bearing a strong inverse association with survival.^3^ The authors considered that the M/I ratio for a specific cancer, “*taken in conjunction with known average survival rates*, should thus give some indication as to the completeness of registration.”^3(p49)^ It is important to note that the M/I ratio was not being proposed as a surrogate for cancer survival. On the contrary, its interpretation as a measure of the completeness of cancer registration data required local knowledge of cancer survival in the first place.

The International Agency for Research on Cancer manual on cancer registration,^4^ published in 1991, went further, suggesting that if cancer registries were unable to estimate survival directly by comprehensive follow-up of all patients with cancer who have been registered to determine their vital status, then the M/I ratio, which was incorrectly described as the fatality ratio, could be used instead as an indicator of survival (duration unspecified). Importantly, however, the manual did acknowledge that the patients who had been registered with cancer and the persons certified as having died of cancer were not the same persons, so the M/I ratio was only “an indirect description of the general survival experience.”^4(pp175-176)^ However, the complement of the M/I ratio (1-M/I ratio) is now increasingly often presented as if it were a valid surrogate for cancer survival, typically for 5-year survival, although the duration of survival is often unstated. This use of the M/I ratio is mistaken in principle and misleading in practice.

We discuss why the 1-M/I ratio cannot be safely used as a proxy for cancer survival, for any cancer, or at any time since diagnosis. We also assess the extent to which levels and trends in the 1-M/I ratio reflect trends in cancer survival at 1, 5 and 10 years after diagnosis. To do this, we use the national cancer incidence, mortality and survival data for 19 cancers in men and 20 cancers in women in England for each of the 29 years from 1981 to 2009, to compare trends in net survival for each cancer with trends in the 1-M/I ratio for that cancer.

## CANCER INCIDENCE AND SURVIVAL

Cancer incidence and mortality rates summarize the numbers of new diagnoses and of deaths attributed to cancer, respectively, that have arisen in a given year, per 100,000 population. By contrast, a cancer survival estimate is not a rate. It is the cumulative probability of survival up to some specified time since diagnosis, such as 5 years, among a specified cohort of people who have all been diagnosed with cancer. In survival studies, all persons are exposed to the risk of death from cancer because they have been diagnosed with it. By contrast, again, most persons in the general population are not exposed to the risk of death from cancer, because they have not been diagnosed with it. This may seem obvious, but it is crucial in understanding the distinction between a cancer mortality rate and a cancer survival estimate.^1^

The probability of survival of a cohort of patients with cancer declines with time since diagnosis; therefore, a cancer survival estimate is only useful if the time since diagnosis is specified. By contrast, the M/I ratio for a given year is a single number, unrelated to the time since diagnosis for any patient. It does not reflect the survival experience of patients with cancer diagnosed in any year.

The 1-M/I ratio is not a valid proxy for cancer survival for several reasons: (1) the mortality rate does not refer to the same patients as the incidence rate, (2) the validity of cancer mortality rates may be questionable, (3) the cause of death stated on death certificates is less precise than the diagnosis recorded in cancer registry records, and (4) there is no mathematical relationship between the M/I ratio and survival. These points are discussed in detail in the following sections.

### The Mortality Rate Does Not Refer to the Same Patients as the Incidence Rate

A cancer incidence rate refers to patients who were diagnosed in a given year. By contrast, a cancer mortality rate refers to people who died in that year and for whom the death certificate mentioned a cancer that was subsequently coded as the underlying cause of death. The date of a cancer diagnosis is not recorded on the death certificate. For the persons who were certified as having died of cancer in any given year, the unknown dates of diagnosis are backscattered through time to an unknown extent, which depends on past trends in survival. The higher the survival, the further back in time the dates of diagnosis will be scattered. For example, women with breast cancer who died in 2009 could have been diagnosed with the disease as far back as 1994 or even earlier.^1^

The M/I ratio makes no allowance for this. Any rapid change in survival will only be reflected in the M/I ratio when the interval between diagnosis and death has elapsed, and this interval will vary over time as survival improves. For cancers with high survival, this interval is long. Conversely, for a very lethal cancer, the mortality rate in a given year largely relates to the same group of patients as the incidence rate; therefore, a change in prognosis will be reflected in the M/I ratio more rapidly. The intrinsic variability between cancers of different lethality in the responsiveness of the M/I ratio to changes in survival, and to the level of survival at any time since diagnosis, invalidates the 1-M/I ratio as a survival metric that would be robust for all cancers, all countries, all calendar periods of diagnosis, or any particular time since diagnosis.

### The Validity of Cancer Mortality Rates May Be Questionable

The validity and international comparability of cancer mortality rates depend on three factors: (1) the completeness of death registration, (2) the accuracy of certification of the cause(s) of death, and (3) if more than one cause of death has been certified in the causal sequence, the accuracy of selection and coding of the underlying cause of death. The underlying cause of death is what determines whether the death is included in the numerator of the mortality rate for that condition (cancer or some other cause). In most high-income countries, death certification is statutory, civil registration systems are efficient, and virtually every death is registered. Inaccuracy in mortality rates can still arise from errors in certification of the cause(s) of death or inconsistencies between the doctors who complete the death certificate. The underlying cause of death may also be incorrectly selected and coded, unless automated systems are used.^5-7^

The 1-M/I ratio was originally suggested as a useful proxy for survival only when population-based survival estimates were unavailable.^4^ Today, that constraint applies mainly to low- and middle-income countries, where death certification may be incomplete. According to the WHO, two thirds of the 56 million deaths that occur every year are not even registered.^8^ Only 64 of the 115 WHO Member States reporting mortality data in 2003 had high-quality vital registration that also included certification of the cause of death.^9^ This is largely due to insufficient resources for universal death registration and medical certification of the cause(s) of death.^10^ Only one third of countries outside North America and Europe can produce usable mortality statistics. Half the countries in Africa and Southeast Asia do not record the cause of death.^11^

Conversely, in some low-resource countries, cancer registration and the updating of vital status is remarkably complete, because information is collected through household visits by local field staff. If cancer incidence data are more complete than the mortality data for the general population, the M/I ratio will reflect incompleteness of mortality data, not the completeness of the incidence data. For example, population-based registries in India routinely report M/I ratios for all cancers combined in the range of 15% to 30%.^12^ This is not likely to represent 5-year survival for all cancers combined in the 70% to 85% range.

### The Cause of Death Stated on Death Certificates Is Less Precise Than the Diagnosis Recorded in Cancer Registry Records

A population-based cancer registry will usually have more precise information on a cancer diagnosis that was made during life than a diagnosis based solely on what is written on the death certificate. The underlying cause of death is therefore frequently coded to a less specific disease category than in the cancer registry.^2^ A cancer mortality rate, derived from death certificates, may then refer to a broader category of disease than the cancer incidence rate with which it would be compared in the M/I ratio. Misclassification of the cause of death can complicate estimation of cause-specific survival, even for common cancers.^13^ For this reason, some cancer registries recode the underlying cause of death provided by the national statistics office, using diagnostic information from the medical record for each patient.^14^

One US study found that even for people known to have been diagnosed with cancer, the underlying cause of death on the death certificate only matched the cause of death recorded by the cancer registry for approximately 80% of patients (all cancers combined). The concordance varied dramatically between cancers, from 10% to 95%.^15^ Agreement was better for the most common cancers, but considerable misclassification was seen for less common cancers.

### There Is No Mathematical Relationship Between the M/I Ratio and Survival

There is a clearly defined mathematical relationship between population-based measures of cancer incidence, survival, and mortality.^1^ By contrast, to the best of our knowledge, no mathematical relationship exists between the M/I ratio and survival, regardless of the duration of time since diagnosis at which survival is to be estimated. For some cancers, the 1-M/I ratio may sometimes be numerically similar to 5-year net survival, but this similarity does not hold for other time intervals of survival since diagnosis, or for all cancers, or even for the same cancer over calendar time. In the absence of a defined mathematical relationship between the M/I ratio and survival, the 1-M/I ratio is not defensible as a surrogate for cancer survival at any time since diagnosis.

We now examine the relationship between the 1-M/I ratio and survival empirically. We used data on cancer incidence, mortality, and survival in England, where the registration of cancers and deaths is longstanding, of high quality, and considered complete.

## METHODS

For 19 cancers in men and 20 cancers in women (39 cancer-sex combinations), we compared trends in the incidence rate, mortality rate, net survival probability at 1, 5, and 10 years after diagnosis, and 1-M/I ratio for patients diagnosed in England in each of the 29 years from 1981 to 2009. Individual tumor registrations for newly diagnosed patients were obtained from the National Cancer Registry. Individual death records were obtained from the national mortality database at the Office for National Statistics. Annual incidence and mortality rates were calculated for each cancer, sex, and 5-year age group. The M/I ratio was calculated from incidence and mortality rates for all ages combined, age standardized to the 2013 European Standard Population.^16^

The vital status of all patients registered with a cancer was ascertained up to December 31, 2013. Net survival up to 10 years after diagnosis was estimated for each of the 39 cancer-sex combinations. We used a flexible excess hazard regression model in which the year of diagnosis and age were modeled as quantitative variables.^17^ Net survival can be interpreted as survival from the cancer of interest, after correction for the hazard of death from competing causes (background mortality).^18,19^ Background mortality is taken from general population life tables of all-cause death rates, defined by single year of age, sex, and calendar year. This is crucial in the comparison of survival estimates between countries, or over time in the same country, because background mortality varies widely by age and sex, as well as between countries and over time. Where fewer than 10 years of follow-up were available, the model was used to predict net survival up to 10 years after diagnosis. Cancer survival estimates for all ages combined were age standardized with the International Cancer Survival Standard weights.^20^

The cancer incidence rates and cancer survival probabilities for each calendar year from 1981 to 2009 refer to the patients who were diagnosed in that year. The cancer mortality rates are presented for people certified as having died from cancer in that year. Trends in age-standardized net survival and in the 1-M/I ratio are presented alongside trends in the age-standardized incidence and mortality rates for selected cancers.

## RESULTS

The absolute difference between the 1-M/I ratio and 5-year net survival for 2009 was at least 5% for 27 of the 39 cancer-sex combinations examined. For 12 of these 39 cancer-sex combinations, the difference was 15% or more: stomach, colon, liver, bladder, and brain in both sexes; cervical cancer; and rectum in men (Table 1). For many cancers, the difference between the two statistics varied between men and women.

**TABLE 1:**
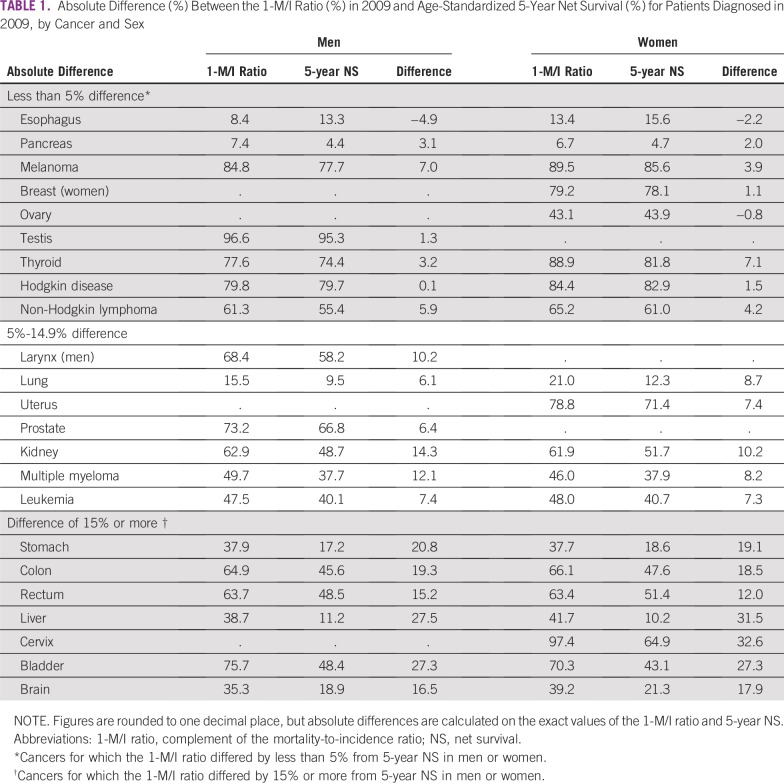
Absolute Difference (%) Between the 1-M/I Ratio (%) in 2009 and Age-Standardized 5-Year Net Survival (%) for Patients Diagnosed in 2009, by Cancer and Sex

### Five-Year Net Survival and the 1-M/I Ratio Differed by Less Than 5%

**FIG 1:**
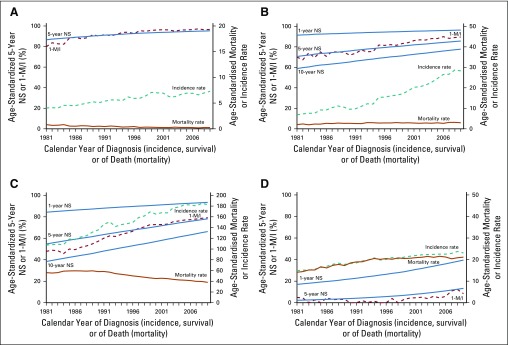
Trends in age-standardized incidence, mortality, complement to mortality-to-incidence ratio (1-M/I ratio), and net survival (NS) in England, 1981 to 2009. Four cancers for which the 1-M/I ratio for 2009 differed by less than 5% from 5-year NS for patients diagnosed in 2009: (A) testicular; (B) melanoma of the skin (women), (C) breast (women), and (D) esophagus (men). Solid blue lines, 1-, 5-, and 10-year NS; dashed brown line, 1-M/I; dashed teal line, incidence rate; solid brown line, mortality rate.

The difference between the 1-M/I ratio and 5-year net survival for patients diagnosed in 2009 was less than 5% for 12 cancer-sex combinations: esophagus, pancreas, and Hodgkin disease in both sexes; melanoma, breast, ovary and non-Hodgkin lymphoma in women; and testis and thyroid in men. Most of these cancers have a good prognosis. Testicular cancer has the highest survival of all common cancers in men (5-year net survival in 2009, 95%), and the 1-M/I ratio tracks the trend in 5-year net survival closely (Fig 1A). Among women, melanoma of the skin has the highest survival of all common cancers (5-year net survival in 2009, 86%). From the early 1980s to the mid-1990s, the 1-M/I ratio for melanoma in women was similar to 5-year net survival, but the trends in the 1-M/I ratio and 5-year net survival start to diverge around 1995, after a distinct increase in incidence (Fig 1B).

Five-year net survival among women diagnosed with breast cancer was also high (78% in 2009), and from the late 1990s onward, the 1-M/I ratio was almost identical to 5-year net survival (Fig 1C). Before then, however, trends in the 1-M/I ratio and 5-year net survival were widely divergent. For example, the 1-M/I ratio for breast cancer in 1984 was almost 20% lower than the 5-year net survival estimate for women diagnosed with breast cancer in that year.

Five-year net survival among men diagnosed with esophageal cancer was just 13% in 2009. Despite some fluctuation, the trend in the 1-M/I ratio largely tracked the trend in 5-year net survival (Fig 1D). Among the cancers we examined, esophageal cancer was the only one for which the 1-M/I ratio was consistently lower than 5-year net survival.

### Five-Year Net Survival and the1-M/I Ratio Differed by 15% or More

**FIG 2:**
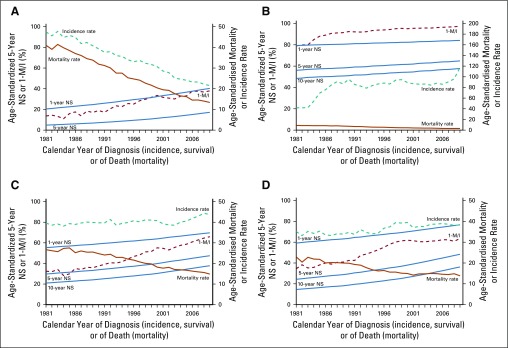
Trends in age-standardized incidence, mortality, complement to mortality-to-incidence ratio (1-M/I ratio), and net survival (NS) in England, 1981 to 2009. Four cancers for which the 1-M/I ratio for 2009 differed by 15% or more from 5-year NS for patients diagnosed in 2009: (A) stomach (men), (B) cervix, (C), colon (women), and (D) rectum (men). Solid blue lines, 1-, 5-, and 10-year NS; dashed brown line, 1-M/I; dashed teal line, incidence rate; solid brown line, mortality rate.

For stomach cancer, trends in incidence, mortality, and survival changed dramatically from 1981 to 2009. The 1-M/I ratio was consistently much higher than 5-year net survival for both men (by 21% in 2009; Fig 2A) and women (by 19% in 2009; Data Supplement).

The incidence of cervical cancer also changed considerably over the 29-year period from 1981 to 2009. Although the trends in the 1-M/I ratio and 5-year net survival followed a broadly similar pattern, the difference in level between the 1-M/I ratio for 2009 and the 5-year net survival estimate for women diagnosed in 2009 was as much as 32% (Fig 2B).

For cancers of the colon and rectum, mortality declined and survival improved from 1981 to 2009. For both cancers, the trend in the 1-M/I ratio roughly tracked 5-year net survival until the early 1990s, but the trends then became increasingly divergent. By 2009, the 1-M/I ratio was 19% higher than 5-year net survival for colon cancer in both men and women (Fig 2C; women), and 12% to 15% higher than 5-year net survival for rectal cancer (Fig 2D; men).

For liver cancer, the trends in incidence, mortality, 1-M/I ratio, and 5-year net survival are widely discordant. Changes in the diagnosis and recording of brain tumors,^21^ and changes in the definition of what is recorded as an invasive bladder cancer also make it difficult to interpret trends in incidence and survival for these cancers in England.^22^ Trends in the 1-M/I ratio are vastly different from the trends in 5-year net survival for cancers of the liver, brain, and bladder (Data Supplement).

## DISCUSSION

For most cancers, the difference between 5-year net survival and the 1-M/I ratio in England changed dramatically between 1981 and 2009. Our analyses of trends in cancer incidence, mortality and survival in England show that, for more than half of 19 common cancers in men and 20 common cancers in women, the 1-M/I ratio is not even closely similar to the estimates of 5-year net survival. The starkest examples are for stomach cancer, for which incidence and mortality rates have declined steadily, and cervical cancer, for which incidence was increasing but has fluctuated widely. The results also show that the difference between the 1-M/I ratio and 5-year net survival for any given cancer can change dramatically over time.

For some cancers, changes or inaccuracy in the diagnosis, recording, and coding of tumors in the cancer registry, and on death certificates, make it difficult to interpret trends in incidence, mortality, and survival. Our analyses raise concern about the quality of mortality data for liver cancer. This observation is not new; in one US study, only 53% of deaths among patients with microscopically confirmed primary liver cancer were recorded with primary liver cancer as the underlying cause of death on the death certificate.^23^

The 1-M/I ratio was initially suggested in the 1970s as an indicator of the completeness of population-based cancer registries. In the early 1990s, it was suggested as a proxy indicator of 5-year survival, but only for cancer registries that were unable to compute survival from individual records for patients with cancer because of a lack of comprehensive follow-up.^4^ The authors even cautioned that it was not clear whether M/I ratios could provide any meaningful comparison of survival between cancer registries.

Unfortunately, the caution expressed by the authors of the cancer registration manual has been widely ignored. We argue that, in fact, such comparisons cannot be safely made.

The evidence we present shows that the 1-M/I ratio is not a reliable proxy for 5-year net survival, still less for survival at 1 year or 10 years after diagnosis. The difference between 5-year survival and the 1-M/I ratio varies considerably between cancers, between men and women, between populations, and for any given cancer, over time. The completeness of death registration and the accuracy of selection and coding of the underlying cause of death are often problematic, especially in low- and middle-income countries. Above all, cancer incidence and mortality rates for a given year relate to different cohorts of people.

The M/I ratio is not an estimate of the case-fatality ratio, which is the proportion of persons diagnosed with a cancer who die within a specified period of time, usually within a few weeks or months. Recently, the 1-M/I ratio has been formally evaluated as a possible proxy for 5-year relative survival from publicly available data on incidence, mortality, and survival for 32 cancers in the Netherlands, the United States, and the five Nordic countries.^24^ This is curious, because high-quality population-based survival estimates have been routinely published in all those countries for many years. The authors commented that the 1-M/I ratio was a close approximation of 5-year relative survival for some cancers, but that it was not a valid proxy for all cancers. They also noted that the degree to which the 1-M/I ratio deviated from 5-year survival was random. They nevertheless concluded that “[t]he 1-(M/I) is a good approximation of the 5-year relative survival for most but not all tumour sites.”^24(p573)^

That argument is hard to follow. The inconsistency of the results, both between cancers and between countries, does not support the use of the 1-M/I ratio as a valid proxy for 5-year survival for any cancer, whether to examine trends in survival in a given country or international differences in survival.

Despite this and the cautions expressed by others,^4,25^ the 1-M/I ratio has increasingly been used as a proxy for survival for many cancers, usually without any acknowledgment of its limitations, even when population-based survival data are available from the same area. This is most apparent in the literature on health disparities, where the M/I ratio has been presented as evidence of cancer survival disparities by race,^26-28^ place of birth,^29^ or sex,^26,30^ and even as an indicator of international disparities in cancer screening and health care.^30-34^ Other authors have criticized this use.^35^ One comparison of the 1-M/I ratio with 5-year observed survival from Lima, Peru, mentioned the absence of studies “using data from population-based cancer registries in developing countries to estimate survival,”^36(p799)^ although several such studies have been published, one of which included data and participation from Lima.^37-40^

The M/I ratio has also been misused in high-level reviews. A widely cited report by the Economist Intelligence Unit from 2009 incorrectly referred to the M/I ratio as the “case-fatality ratio” and the “case-fatality rate,” and then asserted, also incorrectly, that its complement is the survival [rate].^41^ Similarly, in *Disease Control Priorities*: *Cancer*, published by the World Bank in 2015, the M/I ratio is incorrectly presented both as an approximation of the percentage of people who die of the disease and of the cancer-specific mortality rate, for which the unit of measurement is not even a percentage.^42^ Most recently, an examination of the burden of cancer in India within the Global Burden of Disease Study deployed the M/I ratio to estimate cancer prevalence as a surrogate for access to cancer care.^43^ These basic errors in major publications on cancer control lead to confusion, and to misinterpretation of the epidemiologic evidence on cancer survival.

Population-based survival estimates from cancer registry data summarize the observed duration of survival of individual patients with cancer. That is what we want to know—the probability of survival among a cohort of patients with cancer who were diagnosed in a given country or region at a given point in time. These estimates allow us to examine the decline in survival with time since diagnosis (survival curves). They enable us to examine variation in survival with age at diagnosis, between the sexes, by stage at diagnosis, by socioeconomic status, by race/ethnicity, and between geographic regions, as well as trends in survival over calendar time, and international differences in survival.

Net survival methods enable us to correct for differences in background mortality (death from causes other than cancer) when comparing cancer survival between populations or over time.^18,44^ We can also derive public health measures, such as statistical cure^45-47^ or the number of avoidable premature deaths among patients with cancer^48-50^ These measures are used to influence cancer policy and to evaluate the effectiveness of health systems in cancer management.^51^

None of this is possible with the 1-M/I ratio. The 1-M/I ratio is not an observation of the survival of a cohort of patients with cancer. It should not be used as an estimate (or a proxy) of cancer survival.

## References

[1] Ellis L Woods LM Estève J et al Cancer incidence, survival and mortality: Explaining the concepts Int J Cancer 135 1774 1782 2014 24945976 10.1002/ijc.28990

[2] Waterhouse JAH Muir CS Correa P et al Cancer Incidence in Five Continents Volume III (IARC Scientific Publications No. 15) Lyon, France International Agency for Research on Cancer 1976

[3] Parkin DM Muir CS Whelan SL et al Cancer Incidence in Five Continents Volume VI (IARC Scientific Publications No. 120) Lyon, France International Agency for Research on Cancer 1993

[4] Jensen OM Parkin DM MacLennan R et al Cancer Registration: Principles and Methods (IARC Scientific Publications No. 95) Lyon, France International Agency for Research on Cancer 1991

[5] Percy C Stanek E III Gloeckler L Accuracy of cancer death certificates and its effect on cancer mortality statistics Am J Public Health 71 242 250 1981 7468855 10.2105/ajph.71.3.242 PMC1619811

[6] Percy C Dolman A Comparison of the coding of death certificates related to cancer in seven countries Public Health Rep 93 335 350 1978 684145 PMC1431921

[7] Laurenti R Coleman MP Aylin P Accuracy of statements of the cause of death on death certificates and the international comparability of mortality statistics, in Coleman MP, Aylin P (eds): Death Certification and Mortality Statistics: An International Perspective. London, United Kingdom, Office for National Statistics; 2000: 1-9

[8] World Health Organisation Civil registration: Why counting births and deaths is important. (Fact Sheet No. 324) http://www.who.int/mediacentre/factsheets/fs324/en/

[9] World Health Organisation WHO mortality database, 2018 http://www.who.int/healthinfo/mortality_data/en/

[10] Jha P Reliable direct measurement of causes of death in low- and middle-income countries BMC Med 12 19 2014 24495839 10.1186/1741-7015-12-19 PMC3912491

[11] Setel PW Macfarlane SB Szreter S et al A scandal of invisibility: Making everyone count by counting everyone Lancet 370 1569 1577 2007 17992727 10.1016/S0140-6736(07)61307-5

[12] National Centre for Disease Informatics and Research: Three-year report of population-based cancer registries 2012-2014. Report of 27 PBCRs in India. NCDIR, New Delhi, India, 2016. http://www.ncdirindia.org/NCRP/all_ncrp_reports/pbcr_report_2012_2014/all_content/pdf_printed_version/preliminary_pages_printed.pdf

[13] Yin D Morris CR Bates JH et al Effect of misclassified underlying cause of death on survival estimates of colon and rectal cancer J Natl Cancer Inst 103 1130 1133 2011 21697545 10.1093/jnci/djr207

[14] Schaffar R Rapiti E Rachet B et al Accuracy of cause of death data routinely recorded in a population-based cancer registry: Impact on cause-specific survival and validation using the Geneva Cancer Registry BMC Cancer 13 609 2013 24373194 10.1186/1471-2407-13-609 PMC3879655

[15] German RR Fink AK Heron M et al The accuracy of cancer mortality statistics based on death certificates in the United States Cancer Epidemiol 35 126 131 2011 20952269 10.1016/j.canep.2010.09.005

[16] EUROSTAT Revision of the European Standard Population. Report of Eurostat’s task force https://ec.europa.eu/eurostat/documents/3859598/5926869/KS-RA-13-028-EN.PDF/e713fa79-1add-44e8-b23d-5e8fa09b3f8f

[17] Lambert PC Royston P Further development of flexible parametric models for survival analysis Stata J 9 265 290 2009

[18] Pohar Perme M Stare J Estève J On estimation in relative survival Biometrics 68 113 120 2012 21689081 10.1111/j.1541-0420.2011.01640.x

[19] Estève J Benhamou E Raymond L Statistical Methods in Cancer Research Volume IV Descriptive Epidemiology (IARC Scientific Publication No. 128) Lyon, France, International Agency for Research on Cancer, 1994 7698823

[20] Corazziari I Quinn M Capocaccia R Standard cancer patient population for age standardising survival ratios Eur J Cancer 40 2307 2316 2004 15454257 10.1016/j.ejca.2004.07.002

[21] Rachet B Mitry E Quinn MJ et al Survival from brain tumours in England and Wales up to 2001 Br J Cancer 99 S98 S101 2008 S1 suppl 1 18813276 10.1038/sj.bjc.6604603 PMC2557537

[22] Shah A Rachet B Mitry E et al Survival from bladder cancer in England and Wales up to 2001 Br J Cancer 99 S86 S89 2008 S1 suppl 1 18813272 10.1038/sj.bjc.6604599 PMC2557536

[23] Percy C Ries LG Van Holten VD The accuracy of liver cancer as the underlying cause of death on death certificates Public Health Rep 105 361 367 1990 2116637 PMC1580081

[24] Asadzadeh Vostakolaei F Karim-Kos HE Janssen-Heijnen MLG et al The validity of the mortality to incidence ratio as a proxy for site-specific cancer survival Eur J Public Health 21 573 577 2011 20813895 10.1093/eurpub/ckq120

[25] Parkin DM Bray F Evaluation of data quality in the cancer registry: Principles and methods Part II. Completeness Eur J Cancer 45 756 764 2009 19128954 10.1016/j.ejca.2008.11.033

[26] Hébert JR Daguise VG Hurley DM et al Mapping cancer mortality-to-incidence ratios to illustrate racial and sex disparities in a high-risk population Cancer 115 2539 2552 2009 19296515 10.1002/cncr.24270 PMC2688832

[27] Babatunde OA Adams SA Eberth JM et al Racial disparities in endometrial cancer mortality-to-incidence ratios among blacks and whites in South Carolina Cancer Causes Control 27 503 511 2016 26830900 10.1007/s10552-016-0724-7

[28] Wagner SE Hurley DM Hébert JR et al Cancer mortality-to-incidence ratios in Georgia: Describing racial cancer disparities and potential geographic determinants Cancer 118 4032 4045 2012 22294294 10.1002/cncr.26728 PMC3342438

[29] Feletto E Sitas F Quantifying disparities in cancer incidence and mortality of Australian residents of New South Wales (NSW) by place of birth: An ecological study BMC Public Health 15 823 2015 26306859 10.1186/s12889-015-2141-3 PMC4548689

[30] Wang SC Sung WW Kao YL et al The gender difference and mortality-to-incidence ratio relate to health care disparities in bladder cancer: National estimates from 33 countries Sci Rep 7 4360 2017 28659584 10.1038/s41598-017-04083-z PMC5489533

[31] Sunkara V Hébert JR The colorectal cancer mortality-to-incidence ratio as an indicator of global cancer screening and care Cancer 121 1563 1569 2015 25572676 10.1002/cncr.29228 PMC4424055

[32] Sunkara V Hébert JR The colorectal cancer mortality-to-incidence ratio as a potential cancer surveillance measure in Asia Asian Pac J Cancer Prev 17 4323 4326 2016 27797238

[33] Lee HL Peng CM Huang CY et al Is mortality-to-incidence ratio associated with health disparity in pancreatic cancer? A cross-sectional database analysis of 57 countries BMJ Open 8 e020618 2018 10.1136/bmjopen-2017-020618 PMC6042615 29982202

[34] Choi E Lee S Nhung BC et al Cancer mortality-to-incidence ratio as an indicator of cancer management outcomes in Organization for Economic Cooperation and Development countries Epidemiol Health 39 e2017006 2017 28171715 10.4178/epih.e2017006 PMC5434228

[35] Cordero-Morales A Savitzky MJ Stenning-Persivale K et al Conceptual considerations and methodological recommendations for the use of the mortality-to-incidence ratio in time-lagged, ecological-level analysis for public health systems-oriented cancer research Cancer 122 486 487 2016 26484751 10.1002/cncr.29747

[36] Stenning-Persivale K Franco MJS Cordero-Morales A et al The mortality-incidence ratio as an indicator of five-year cancer survival in metropolitan Lima Ecancermedicalscience 12 799 2018 29456616 10.3332/ecancer.2018.799 PMC5813917

[37] Sankaranarayanan R Black RJ Parkin DM Cancer Survival in Developing Countries (IARC Scientific Publications No. 145). Lyon, France, International Agency for Research on Cancer, 1998 10194635

[38] Coleman MP Quaresma M Berrino F et al Cancer survival in five continents: A worldwide population-based study (CONCORD) Lancet Oncol 9 730 756 2008 18639491 10.1016/S1470-2045(08)70179-7

[39] Allemani C Weir HK Carreira H et al Global surveillance of cancer survival 1995-2009: Analysis of individual data for 25,676,887 patients from 279 population-based registries in 67 countries (CONCORD-2) Lancet 385 977 1010 2015 25467588 10.1016/S0140-6736(14)62038-9 PMC4588097

[40] Allemani C Matsuda T Di Carlo V et al Global surveillance of trends in cancer survival 2000–14 (CONCORD-3): Analysis of individual records for 37,513,025 patients diagnosed with one of 18 cancers from 322 population-based registries in 71 countries Lancet 391 1023 1075 2018 29395269 10.1016/S0140-6736(17)33326-3 PMC5879496

[41] Beaulieu N Bloom DE Bloom LR et al Breakaway: The global burden of cancer - challenges and opportunities. A report from the Economist Intelligence Unit. London, United Kingdom, The Economist, 2009

[42] Gelband H Jha P Sankaranarayanan R et al Disease Control Priorities: Cancer (ed 3). Washington, DC, International Bank for Reconstruction and Development and The World Bank, 2015 26913318

[43] Dhillon PK Mathur P Nandakumar A et al I The burden of cancers and their variations across the states of India: The Global Burden of Disease Study 1990-2016 Lancet Oncol 19 1289 1306 2018 30219626 10.1016/S1470-2045(18)30447-9 PMC6167407

[44] Ederer F Axtell LM Cutler SJ The relative survival rate: A statistical methodology Natl Cancer Inst Monogr 6 101 121 1961 13889176

[45] Binbing Y Tiwari RC Feuer EJ Estimating the personal cure rate of cancer patients using population-based grouped cancer survival data Stat Methods Med Res 20 261 274 2011 20181780 10.1177/0962280209347046 PMC2888754

[46] Seppä K Hakulinen T Kim H-J et al Cure fraction model with random effects for regional variation in cancer survival Stat Med 29 2781 2793 2010 20862662 10.1002/sim.4046

[47] Lambert PC Thompson JR Weston CL et al Estimating and modeling the cure fraction in population-based cancer survival analysis Biostatistics 8 576 594 2007 17021277 10.1093/biostatistics/kxl030

[48] Sandiford P Abdel-Rahman ME Allemani C et al How many cancer deaths could New Zealand avoid if five-year relative survival ratios were the same as in Australia? Aust N Z J Public Health 39 157 161 2015 25716332 10.1111/1753-6405.12344

[49] Ellis L Coleman MP Rachet B How many deaths would be avoidable if socioeconomic inequalities in cancer survival in England were eliminated? A national population-based study, 1996-2006 Eur J Cancer 48 270 278 2012 22093945 10.1016/j.ejca.2011.10.008

[50] Abdel-Rahman M Stockton D Rachet B et al What if cancer survival in Britain were the same as in Europe: How many deaths are avoidable? Br J Cancer 101 S115 S124 2009 S2 suppl 2 19956155 10.1038/sj.bjc.6605401 PMC2790713

[51] Organisation for Economic Co-operation and Development Health at a Glance 2017: OECD indicators Paris, France OECD Publishing 2017

